# DtxR is a global iron-dependent regulatory protein with multiple roles in the control of gene expression in *Corynebacterium diphtheriae*

**DOI:** 10.1128/jb.00530-25

**Published:** 2026-02-26

**Authors:** Lindsey R. Lyman, Eric D. Peng, Michael P. Schmitt

**Affiliations:** 1 Laboratory of Respiratory and Special Pathogens, Division of Bacterial, Parasitic, and Allergenic Products, Center for Biologics Evaluation and Research, Food and Drug Administration, Silver Spring, Maryland, USA; University of Illinois Chicago, Chicago, Illinois, USA

**Keywords:** DtxR, iron, *Corynebacterium*

## Abstract

*Corynebacterium diphtheriae* is the causative agent of diphtheria, a severe respiratory disease in humans. Diphtheria toxin, encoded by the *tox* gene, is a potent exotoxin secreted by *C. diphtheriae* that is responsible for much of the morbidity and mortality of diphtheria. Expression of the *tox* gene is regulated by iron and the diphtheria toxin repressor (DtxR). In addition to controlling toxin expression, DtxR functions as a global iron-dependent regulatory factor that mediates iron homeostasis in *C. diphtheriae*. While numerous genes regulated by DtxR and iron are known, a genome-wide study of both the iron and DtxR regulons is lacking in *C. diphtheriae*. Here, we report novel iron- and DtxR-regulated genes revealed by a comprehensive transcriptomic analysis in *C. diphtheriae*. The findings from the study show that iron and DtxR not only result in the repression of transcription of numerous genes but also induce gene expression. Some of the genes induced by iron and DtxR were found to be regulated by the RipA repressor, which is encoded by the *ripA* gene whose transcription is repressed by iron and DtxR. Electrophoretic mobility shift assays confirmed DtxR binding to sequences upstream of several of the newly identified genes. Expression of the gene encoding ferritin (*ftn*) was induced by iron and DtxR and required multiple DtxR binding sites upstream of the *ftn* promoter for optimal expression. Collectively, these results expand our knowledge on the function of DtxR and the diverse roles of this regulatory protein in controlling gene expression.

**IMPORTANCE:**

*Corynebacterium diphtheriae* is an important human pathogen that has served as a model system for the study of iron- and diphtheria toxin repressor (DtxR)-gene regulation in gram-positive bacteria. In *C. diphtheriae*, we found that iron and DtxR are involved in the regulation of iron acquisition systems and iron-stress tolerance mechanisms, which are both considered important virulence factors in bacterial pathogens. Iron-regulated genes may encode components to be considered in the formulation of new vaccines or be used as therapeutic targets in the treatment of disease caused by *C. diphtheriae*. The findings in this report also expand our knowledge as to how DtxR functions and the diverse roles of this regulatory protein in controlling gene expression.

## INTRODUCTION

*Corynebacterium diphtheriae* is the etiological agent of diphtheria, a disease associated with respiratory and cutaneous infections in humans. The exotoxin, diphtheria toxin (DT), induces cell death by inhibiting protein synthesis and is responsible for the severe symptoms associated with respiratory diphtheria. Although the gene that encodes DT, *tox*, is found on a prophage locus, the regulation of DT production is dependent upon the availability of iron and diphtheria toxin repressor (DtxR), which is encoded by the *dtxR* gene that resides on the bacterial chromosome (1–4). DT expression is repressed in iron-replete conditions but derepressed under iron limitation. When intracellular iron levels are sufficient, iron binds to DtxR, resulting in conformational changes in the protein that allow DtxR to interact with DNA at a 19-base pair motif that is typically found near promoter elements (5). *C. diphtheriae* DtxR has a broad role in regulating numerous genes associated with iron acquisition and metabolism (6).

Iron is critical for essential processes in bacteria, including nucleotide synthesis (7), oxidative stress tolerance (8), and general metabolism (9). Iron in excess, however, may cause oxidative stress through the generation of reactive free radicals by the Haber-Weiss reaction (10). Organisms must maintain appropriate intracellular iron levels to enable cellular processes to continue while minimizing toxicity. Within the human host, available iron is limited by numerous host factors that sequester iron (11). The host proteins transferrin and lactoferrin bind free iron with high affinity in serum and at mucosal interfaces, restricting iron availability. Bacteria produce siderophores, small molecules with high affinity for iron, to compete against transferrin and lactoferrin iron sequestration (12). However, certain siderophores can be bound by the host protein siderocalin (13, 14), which prevents uptake by bacteria and further limits iron availability. Within eukaryotic cells, iron can be associated with proteins, such as those with iron-sulfur clusters, or complexed to protoporphyrin in the form of heme. Most of the heme in the human host is sequestered by hemoglobin, which serves as a critical source of iron for numerous bacterial pathogens (15).

The mechanisms of iron restriction encountered by bacterial pathogens present challenges that must be overcome to successfully colonize the host and cause disease. *C. diphtheriae* employs numerous mechanisms to acquire heme-iron from the human host, and the genes encoding these heme-iron utilization systems represent a significant portion of the known iron- and DtxR-regulated genes (16–24). *C. diphtheriae* also produces the iron- and DtxR-regulated siderophore corynebactin (25) and its cognate ABC-type transporter (26). Additionally, *C. diphtheriae* encodes several other putative iron or metal ABC-type transporters for which substrates have not been identified (18, 27). While many genes of the DtxR and iron regulons have been described (6, 28), a genome-wide transcriptomic analysis comparing both the iron and DtxR regulons has not been done for *C. diphtheriae*.

In this study, we present a global analysis of differential gene expression in response to iron and DtxR using an iron-limited, semi-defined minimal medium for the growth of a *C. diphtheriae* wild-type (wt) strain and an isogenic *dtxR* mutant. In addition to known iron- and DtxR-repressed genes, we found numerous targets that have not been previously reported. By demonstrating DtxR binding at putative promoter regions for new targets, we show that DtxR not only functions to repress gene transcription but can also directly and indirectly induce gene expression. For example, expression of the gene (*ftn*) encoding the ferritin protein is activated directly through DtxR binding upstream of the *ftn* promoter region under high-iron conditions. We further demonstrate that the RipA protein, whose gene is repressed by DtxR in elevated iron conditions, functions to repress the expression of several genes encoding metabolic proteins in low-iron environments. The findings from this study expand our knowledge of the iron and DtxR regulon in *C. diphtheriae* and reveal that DtxR acts through diverse mechanisms to control gene expression.

## RESULTS

### Identification of differentially expressed genes

We used transcriptome sequencing (RNA sequencing [RNA-seq]) to assess global changes in gene expression in response to iron availability in the *C. diphtheriae* wt strain 1737. Additionally, we examined differential gene expression between the wt 1737 strain and an isogenic *dtxR* point mutant, R47H. The mutation in R47H is a single-nucleotide substitution in the 1737 *dtxR* gene that results in a change from Arg to His at position 47 in the DtxR protein (29). Previous studies with the C7 strain of *C. diphtheriae* carrying the *dtxR* R47H mutation showed that the DtxR-R47H protein was produced by the mutant strain but had significantly reduced binding to the DtxR consensus binding site when compared to wt DtxR (29). The R47H mutation has been extensively examined in previous studies using the C7 strain of *C. diphtheriae* to assess iron- and DtxR-dependent gene expression (16, 19, 29). These earlier studies demonstrated that the R47H amino acid substitution in the DtxR protein resulted in a significant reduction in the ability of DtxR to repress gene expression of several DtxR-repressed genes.

**Fig 1:**
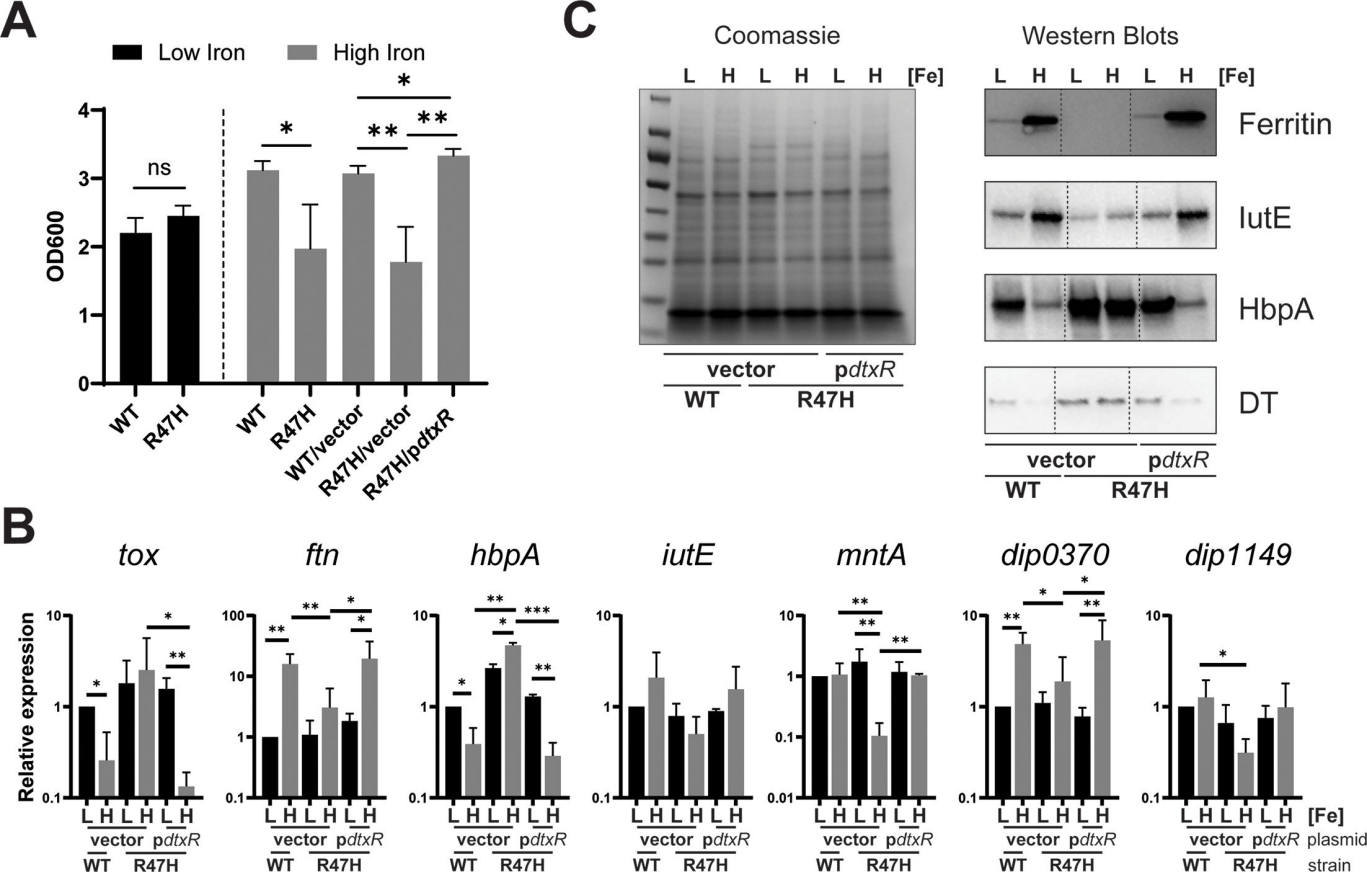
(**A**) Comparison of the growth of wt *C. diphtheriae* strain 1737 and the 1737 *dtxR* mutant, R47H, was assessed in low- (0.25 µM FeCl_3_) and high-iron (5 µM FeCl_3_) mPGT media; plasmids indicated: vector (pKN2.6Z) and p*dtxR* (pKN2.6Z carrying the wt *dtxR* gene). Results show the mean and standard deviation from at least three experiments. **P <0.01; *P <0.05; and ns for not significant by unpaired t test. (**B**) qPCR analysis of indicated target genes from indicated strain/plasmids grown in either low- (L, 0.25 µM) or high- (H, 5 µM) iron conditions. Relative expression is compared to wt under low-iron conditions for each respective gene. Graphs show means with standard deviations of at least three replicates. Unpaired t tests were performed on ΔCq values for the indicated comparisons: ***P <0.001; **P <0.01; and *P <0.05. (**C**) Coomassie-stained SDS gel of cell lysates (left) and a Western blot (right) performed on cell lysates were probed with antibodies specific to the proteins Ferritin, IutE, and HbpA. Culture supernatants containing DT were also tested. Strains carrying either the vector or p*dtxR* were grown with either low (L, 0.25 µM) or high (H, 5 µM) iron. A representative experiment is shown.

For the transcriptomic analysis, RNA was isolated from *C. diphtheriae* grown in iron-depleted mPGT medium that was supplemented with either 0.25 µM (low-iron) or 5 µM (high-iron) FeCl_3_. The mPGT medium used throughout this study was slightly altered from the mPGT medium used in previous studies (30) (see Materials and Methods). The R47H mutant showed similar growth to that of the wt strain in the low-iron mPGT medium but exhibited reduced growth under high-iron conditions, which could be restored to wt growth levels in the presence of the cloned *dtxR* gene (Fig. 1A). To identify genes differentially expressed in response to iron, we compared expression levels in wt *C. diphtheriae* following growth in low- and high-iron mPGT medium. Genes regulated by DtxR were identified by comparing the wt and the R47H mutant following growth in high-iron medium. Genes with a log_2_ fold change greater than 1 and *P* < 0.05 were considered significantly regulated.

In the transcriptomic analysis, we identified 139 genes in which transcription was both iron- and DtxR-regulated. The expression of 73 genes was repressed by iron in a DtxR-dependent manner (Table 1), while 66 genes were induced by iron and DtxR (Table 2). To assess the validity of the RNA-seq analysis, we examined the expression of a representative group of genes using qPCR and Western blots (Fig. 1B and C). Expression of all of the genes examined in Fig. 1B and C showed regulation that was consistent with what was observed in the RNA-seq analysis, and the presence of the cloned wt *dtxR* gene on plasmid p2.6dtxR restored wt gene expression in the R47H mutant (Fig. 1B and C).

**TABLE 1:** Iron- and DtxR-repressed genes

Locus^*a*,^^*b*^	Gene description	Log_2_ fold change^*c*^		
		wt H:L	wt:R47H H:H	R47H H:L
**DIP0014 ↓**	ABC transporter	−5.06	−4.76	
**DIP0015**	Transport system, ATP-binding protein	−4.37	−3.94	
DIP0108 ↓	*irp6A* | Ferrisiderophore receptor	−4.31	−4.19	
DIP0109	*irp6B* | Membrane protein permease	−3.48	−3.78	
DIP0110	*irp6C* | ATP-binding protein	−3.53	−3.25	
**DIP0111**	Putative lipoprotein	−1.27	−1.39	
**DIP0154**	Putative endopeptidase	−3.00	−2.61	
**DIP0155** ↓	Putative secreted protein	−1.15	−1.27	
**DIP0156**	Putative membrane protein	−1.42	−1.30	
**DIP0157**	Putative lipoprotein-ABC transporter	−1.45	−1.63	
DIP0169 ↓	*iutA* | Mn/Zn/Fe substrate-binding protein	−5.89	−5.38	
DIP0170	*iutB* | ATP-binding protein	−4.30	−5.12	1.27
DIP0171	*iutC* | Membrane protein permease	−3.86	−4.65	1.62
DIP0172	*iutD* | Membrane protein permease	−2.16	−2.86	1.51
DIP0222	*tox* | Diphtheria toxin	−2.32	−1.33	
**DIP0321** ↓	ABC transport system ATP-binding protein; (dip0321-0325)^*d*^	−1.21	−1.58	
**DIP0322**	ABC transport system ATP-binding protein	−1.38	−1.51	
**DIP0323**	ABC transport system membrane protein	−1.34	−1.38	
**DIP0406**	Putative membrane protein; DUF418	−2.22	−2.28	
DIP0522	*chtC* | heme-iron transport-associated membrane/secreted protein	−6.78	−7.28	
DIP0523 ↑	*cirA* | putative membrane protein	−6.88	−7.34	
**DIP0570**	Putative membrane protein	−1.37	−2.83	2.22
**DIP0579**	DUF5319 domain-containing protein	−2.63	−2.91	
**DIP0580** ↓	*guaB* | inosine-5'-monophosphate dehydrogenase; (dip0580-0581)^*d*^	−1.00	−1.71	
DIP0582 ↓	*ciuA* | Fe-siderophore (corynebactin) substrate-binding protein	−4.95	−5.04	
DIP0583	*ciuB* | membrane protein permease	−4.30	−4.79	
DIP0584	*ciuC* | membrane protein permease	−3.69	−4.67	1.46
DIP0585	*ciuD* | ATP-binding protein	−4.43	−3.54	
DIP0586 ↓	*ciuE* | siderophore (corynebactin) biosynthesis; (dip0586-dip0588)^*d*^	−6.71	−6.57	
DIP0587	*ciuF* | MFS family; possible role in siderophore secretion	−4.13	−3.43	
**DIP0611**	Putative ABC transport system lipoprotein	−1.38	−4.39	2.85
**DIP0621**	Putative membrane protein	−1.44	−2.50	1.40
**DIP0622**	Putative membrane protein	−2.05	−3.02	1.20
**DIP0623**	*metX* | homoserine O-acetyltransferase	−4.46	−5.20	
DIP0624 ↑	*htaC* | heme-iron transport-associated membrane protein	−8.94	−9.39	
DIP0625 ↓	*htaA* | heme-iron transport-associated membrane/secreted protein	−7.17	−6.83	
DIP0626	*hmuT* | Heme substrate-binding protein	−6.88	−6.28	
DIP0627	*hmuU* | membrane protein	−5.43	−5.13	
DIP0628	*hmuV* | ATP-binding protein	−3.80	−3.38	
DIP0629	*htaB* | heme-iron transport-associated membrane protein	−4.90	−5.02	
**DIP_RS14800**	Transposase	−1.19	−2.74	1.29
**DIP0757**	IS element transposase (partial)	−1.16	−2.95	
**DIP0761**	Cupin type-2 domain-containing protein	−3.11	−2.28	
**DIP0762** ↑	Methyltransferase type 11 domain-containing protein	−5.98	−4.96	
**DIP0836**	Peptidase M23 domain-containing protein	−1.04	−1.08	
DIP0922	*ripA* | HTH-type transcriptional regulator	−7.30	−6.56	
DIP1059	*frgA* | Iron-siderophore uptake system ATP-binding component	−2.56	−2.69	
DIP1060	*frgB* | Iron-siderophore uptake system transmembrane component	−3.00	−2.95	
DIP1061 ↑	*frgC* | Iron-siderophore uptake system transmembrane component	−3.12	−2.91	
DIP1062	*frgD* | ABC transporter substrate-binding protein	−4.10	−4.15	
**DIP1125**	HD domain-containing protein	−1.60	−1.28	
**DIP1510**	*xerC* | Tyrosine recombinase	−2.00	−2.56	
DIP1518	Insertion element DNA-binding protein	−2.71	−2.37	
DIP1519	*chtB |* heme-iron transport associated membrane protein	−6.46	−6.05	
DIP1520 ↑	*chtA |* heme-iron transport associated membrane protein	−4.75	−3.46	−1.32
**DIP1581**	Putative membrane protein	−1.22	−2.36	
**DIP1662**	GntR-family transcriptional regulator	−1.52	−1.06	
DIP1669	*hmuO* | heme oxygenase	−2.72	−5.16	1.82
**DIP1879**	Putative DNA-binding protein	−1.70	−1.65	
**DIP1953**	Putative amino acid export carrier protein	−1.88	−2.28	1.14
**DIP1952**	Pyruvate dehydrogenase	−3.20	−2.66	1.98
**DIP1992**	Na:dicarboxylate symporter family	−1.85	−2.43	1.71
**DIP2076**	Putative membrane protein	−1.25	−2.22	
DIP2160	*sidB* | Modular polyketide synthase; putative metallophore biosynthesis	−2.28	−1.37	−1.41
DIP2161 ↑	*sidA |* Nonribosomal peptide synthase; putative metallophore biosynthesis (dip02161-2158)^*d*^	−2.44	−1.20	−1.20
DIP2162 ↓	*nikA2* | peptide/nickel transport system substrate-binding protein; (dip02162-02165)^*d*^	−1.46	−1.56	
DIP2163	peptide/Ni transport system permease protein	−1.57	−1.66	
DIP2164	peptide/Ni transport system permease protein	−1.52	−1.35	
**DIP2318**	Conserved hypothetical protein (possible ATP/GTP-binding)	−1.68	−2.02	
**DIP2319**	Cu-Zn Superoxide dismutase	−1.63	−1.54	
**DIP2322**	Uncharacterized protein	−1.08	−1.13	
DIP2330	*hbpA* | heme-iron transport associated secreted/membrane protein	−8.71	−9.45	
DIP2356	Conserved membrane protein	−2.56	−2.70	

^*a*^ Bolded genes are newly identified for iron/DtxR regulation.

^*b*^ For operons, arrows indicate the first gene in the operon and direction of transcription. The last significantly regulated gene in each operon is underlined.

^*c*^ Log2 fold change indicated. Compares relative signal intensity between the wt or R47H strains grown in high (H) or low (L) iron as indicated. Negative value indicates increased transcripts detected in the second condition listed; positive value indicates increased transcripts detected in the first condition.

^*d*^ Parentheses indicate a full operon where not all genes in the operon showed significant regulation.

**TABLE 2:** Iron- and DtxR-induced genes

Locus^*a*^	Gene description	Log_2_ fold Change^*b*^		
		wt H:L	wt:R47H H:H	R47H H:L
DIP0033 ↓	*thiG* | thiazole synthase	1.13		
DIP0034	*thiF* | ThiF family adenylyltransferase	1.10	1.30	
DIP0035	*thiD* | bifunctional hydroxymethylpyrimidine kinase/phosphomethylpyrimidine kinase	1.08		
DIP0116	VIT1 family protein; iron transport and storage	5.06	5.18	
DIP0124	*piuB* | PepSY domain-containing protein	2.48	4.14	−3.89
DIP0125	TIGR00730 family Rossman fold protein	1.21	1.52	
DIP0173	*iutE* | Mn/Zn/Fe substrate-binding protein	2.51	2.14	
DIP0299	WhiB family transcriptional regulator	1.40	2.74	−1.73
DIP0303	CRP-like cAMP-activated global transcriptional regulator GlxR	1.46	1.42	−1.14
DIP0357	Alpha-1,6-glucosidase domain-containing protein	1.49	1.15	−1.09
DIP0370 ↓	*sdhC* | Succinate dehydrogenase cytochrome b subunit	3.73	3.14	
DIP0371	Fumarate reductase/succinate dehydrogenase flavoprotein subunit	3.72	2.87	
DIP0372	Succinate dehydrogenase/fumarate reductase iron-sulfur subunit	3.51	2.49	
DIP0373	Hypothetical protein	1.78	1.49	
DIP0400 ↓	*hemA* | glutamyl-tRNA reductase (dip0400-0403)^*c*^	1.35	1.85	
DIP0489 ↓	*sdaC* | serine/threonine transporter	1.65	3.05	−2.92
DIP0490	sdaA | L-serine ammonia-lyase	1.78	2.95	−3.04
DIP0497	*narI |* respiratory nitrate reductase subunit gamma	1.29	1.48	
DIP0498	*narJ* | nitrate reductase molybdenum cofactor assembly chaperone	2.73	2.44	
DIP0499	*narH* | nitrate reductase subunit beta	2.77	2.55	
DIP0500	*narG* | nitrate reductase subunit alpha	2.88	2.35	
DIP0501 ↑	MFS transporter	2.98	2.45	
DIP0672 ↓	Hydrogenase small subunit	2.96	3.00	
DIP0673	Ni-dependent hydrogenase large subunit	2.43	2.50	
DIP0674	Ni/Fe-hydrogenase, b-type cytochrome subunit	1.41	1.57	
DIP0675	HyaD/HybD family hydrogenase maturation endopeptidase	1.00	1.00	
DIP0739 ↓	Methylmalonyl-CoA carboxytransferase subunit 5S	1.55	2.97	−2.40
DIP0740	Acyl-CoA carboxylase subunit beta	1.58	2.99	−2.72
DIP0741	Hypothetical protein	2.10	2.32	−1.67
DIP0742	Biotin/lipoyl-containing protein	1.89	2.19	−1.75
DIP0956 ↓	Peptide ABC transporter substrate-binding protein	1.41	2.47	−1.83
DIP0957	ABC transporter permease	1.15	2.16	−1.51
DIP0958	ABC transporter permease	1.56	1.60	−1.29
DIP0959	dipeptide ABC transporter ATP-binding protein	1.87	2.38	−1.10
DIP0976	GtrA family protein	1.02	1.29	
DIP0980	Amino acid permease	1.26	1.26	
DIP1030 ↓	Lactate dehydrogenase family protein	4.18	3.53	
DIP1031	LutB/LldF family L-lactate oxidation iron-sulfur protein	4.29	3.57	
DIP1032	(Fe-S)-binding protein	4.35	3.74	
DIP1033	L-lactate permease	1.62	1.59	
DIP1123	Zinc ribbon domain-containing protein YjdM	1.95	1.83	
DIP1124 ↑	Biotin synthase BioB	1.96	3.00	−1.50
DIP1142	Amino acid ABC transporter ATP-binding protein	1.70	1.86	
DIP1143	Amino acid ABC transporter permease	1.94	2.47	
DIP1144 ↑	ABC transporter substrate-binding protein	2.06	2.13	
DIP1149	C4-dicarboxylate transporter DcuC	1.47	4.86	−4.36
DIP1198	Anaerobic C4-dicarboxylate transporter	1.45	3.11	−1.36
DIP1231 ↓	Precorrin-3B synthase (dip1231-1233)^*c*^	3.49	3.12	
DIP1252	HigA family addiction module antitoxin	3.81	4.71	
DIP1255	Anaerobic C4-dicarboxylate transporter	2.01	2.68	
DIP1283	*acn* | aconitate hydratase AcnA	3.48	1.79	
DIP1284	TetR/AcrR family transcriptional regulator	1.50		
DIP1389	Dyp-type peroxidase	1.38	1.70	
DIP1390 ↓	Copper chaperone PCu(A)C	2.20	2.18	
DIP1391	Copper resistance CopC family protein	1.65	2.22	
DIP1500	1-deoxy-D-xylulose-5-phosphate reductoisomerase	1.08	1.27	
DIP1502	23S rRNA (adenine(2503)-C (2))-methyltransferase RlmN	2.30	1.49	
DIP1542	Alanine/glycine:cation symporter family protein	1.28	1.36	
DIP1555	Indole-3-glycerol phosphate synthase TrpC	1.24	1.02	
DIP1686	DUF3052 domain-containing protein	1.25	1.61	
DIP1866	F*tn* | ferritin	5.83	6.09	
DIP1898 ↓	Cytochrome ubiquinol oxidase subunit I	5.00	4.03	
DIP1899	Cytochrome d ubiquinol oxidase subunit II	4.11	3.15	
DIP1900	ABC transporter ATP-binding protein/permease	3.91	2.84	
DIP2054	Acetate kinase	1.19	1.31	−1.03
DIP2055 ↑	*pta* | phosphate acetyltransferase	1.16	1.49	−1.53

^*a*^ For operons, arrows indicate the first gene in the operon and direction of transcription. The last significantly regulated gene in each operon is underlined.

^*b*^ Log2 fold change indicated. Compares relative signal intensity between the wt or R47H strains grown in high (H) or low (L) iron as indicated. Negative value indicates increased transcripts detected in the second condition listed; positive value indicates increased transcripts detected in the first condition.

^*c*^ Parentheses indicate a full operon where not all genes in the operon showed significant regulation.

### DtxR- and iron-repressed genes

Forty-two of the genes in Table 1 whose expression is repressed by iron and DtxR were previously identified and characterized (indicated in normal font). The most notable gene in Table 1 is the *tox* gene (*dip0222*), which encodes DT, the primary virulence factor of *C. diphtheriae* and the source for the current diphtheria vaccine (31). Many of the previously identified DtxR- and iron-repressed genes encode proteins involved in the transport of iron. Several of the iron transport proteins are required for the acquisition and utilization of heme-iron (19, 22, 23), while other transporters are involved in the uptake of non-heme iron, which includes proteins involved in the biosynthesis and transport of the *C. diphtheriae* siderophore corynebactin: *ciuABCDE* (*dip0582-586*) (25, 26). Another important iron-repressed gene is *ripA* (*dip0922*), which encodes a regulatory protein that is known in *C. glutamicum* to repress transcription of several genes that encode iron-containing metabolic enzymes (32, 33).

Among the iron- and DtxR-repressed genes in Table 1 are 31 genes that were not previously shown to be regulated by iron and DtxR (indicated in bold). They are predicted to encode a diverse group of proteins, including factors involved in transport (*dip0014-15*, *dip0611*, *dip1581*, *dip0321-323*, *dip2162-64,* and *dip0570*) and gene regulation; *gntR* (*dip1662*) and *arsR* (*dip1879*). The putative transporters encoded by *dip0611* and *dip0570* were also strongly iron regulated in the *dtxR* mutant background (Table 1; R47H, H:L), suggesting that factors in addition to or other than DtxR are involved in their iron-dependent regulation. The specific function of the gene product of many of these novel genes has not been determined.

### DtxR and iron-induced genes

Of the 66 genes whose expression was increased in high-iron conditions in a DtxR-dependent manner (Table 2), only two genes were previously characterized; *iutE* (*dip0173*), which encodes the substrate-binding component of a metal-dependent ABC transporter, and the *acn* gene (*dip1283*), which encodes the enzyme aconitase (34). The *C. diphtheriae acn* gene is also regulated by RipA (34). Although the expression of the *iutE* gene is regulated by DtxR and iron, DtxR does not bind to the sequence upstream of the *iutE* gene, and no region with significant similarity to the consensus DtxR-binding site was found upstream of *iutE*. Additionally, RipA was not involved in *iutE* regulation, and the mechanism as to how *iutE* expression is regulated by DtxR and iron has not been determined. The genes identified in Table 2 represent a diversity of functions, including several genes encoding transport proteins as well as numerous metabolic enzymes. However, the most strongly iron and DtxR-induced genes were *ftn* (*dip1866*), which encodes the iron storage protein ferritin, and *dip0116*, which is predicted to encode a protein from the VIT1 family that includes proteins involved in iron export and intracellular iron storage (35).

Several genes that were induced in high iron in a DtxR-dependent manner were also strongly repressed in high iron in the *dtxR* mutant, suggesting that the iron regulation observed for these genes is not directly dependent on DtxR (Table 2; R47H, H:L). The repression observed for these genes in the *dtxR* mutant under high-iron conditions could be due to other regulators that are responsive to the high intracellular iron levels that would be expected in the *dtxR* mutant grown in high-iron conditions. One unusual gene among this group of iron-induced genes, in which expression appears to be at least partially independent of DtxR, is *piuB* (*dip0124*), which was previously shown to be repressed under high-Mn conditions by the Mn-responsive transcriptional regulator MntR (36). None of the other genes in Table 2 that exhibit iron repression in the *dtxR* mutant are known to be regulated by MntR or Mn, and the mechanism of regulation for these other genes is not known.

### The *piuB* gene is regulated by iron and Mn

Table 3 shows the RNA-seq results of previously identified Mn-regulated genes in *C. diphtheriae*, which includes the *piuB* gene (24). An interesting feature of the expression of these genes is that almost all of them are strongly repressed by iron in the *dtxR* mutant (Table 3, R47H, H:L). Additionally, expression of these Mn-regulated genes shows significantly stronger repression under high-iron conditions in the *dtxR* mutant than the expression levels observed in the wt strain (Table 3, wt:R47H, H:H). A possible reason for the strong repression of the MntR-regulated genes in the *dtxR* mutant under high-iron conditions is likely associated with the high intracellular iron levels that are expected in the *dtxR* mutant under these growth conditions. These high-iron levels may result in the binding of iron to MntR and the subsequent activation of its DNA-binding function that results in repression of the MntR-regulated genes. In support of this possibility, we previously showed that at high-iron levels, MntR can bind *in vitro* to its native DNA-binding sites, which will inhibit transcription of MntR-regulated genes (37).

**TABLE 3:** MntR-regulated genes showing differential expression in the R47H strain

Locus^*a*^	Gene description	Log_2_ fold change^*b*^		
		wt H:L	wt:R47H H:H	R47H H:L
DIP0124	*piuB* | PepSY domain-containing protein	2.48	4.14	−3.89
DIP0615 ↓	*mntA* | Zn/Mn substrate-binding protein-ABC transporter		6.01	−6.02
DIP0616	*mntB* | ATP-binding protein		5.29	−5.36
DIP0617	*mntC* | Membrane protein permease		4.41	−4.13
DIP0618	*mntD* | Membrane protein permease		3.70	−3.08
DIP1923 ↓	*nrdI* | ribonucleotide reductase		5.48	−6.37
DIP1924	*nrdF2* | ribonucleoside-diphosphate reductase beta chain 2		5.40	−6.44
DIP2261	*sodA* | Fe-Mn superoxide dismutase		−2.49	2.45
DIP2262	Oxidoreductase		4.21	−4.43

^*a*^ For operons, arrows indicate the first gene in the operon and direction of transcription. The last significantly regulated gene in each operon is underlined.

^*b*^ Log2 fold change indicated. Compares relative signal intensity between the wt or R47H strains grown in high (H) or low (L) iron as indicated. Negative value indicates increased transcripts detected in the second condition listed; positive value indicates increased transcripts detected in the first condition.

**Fig 2:**
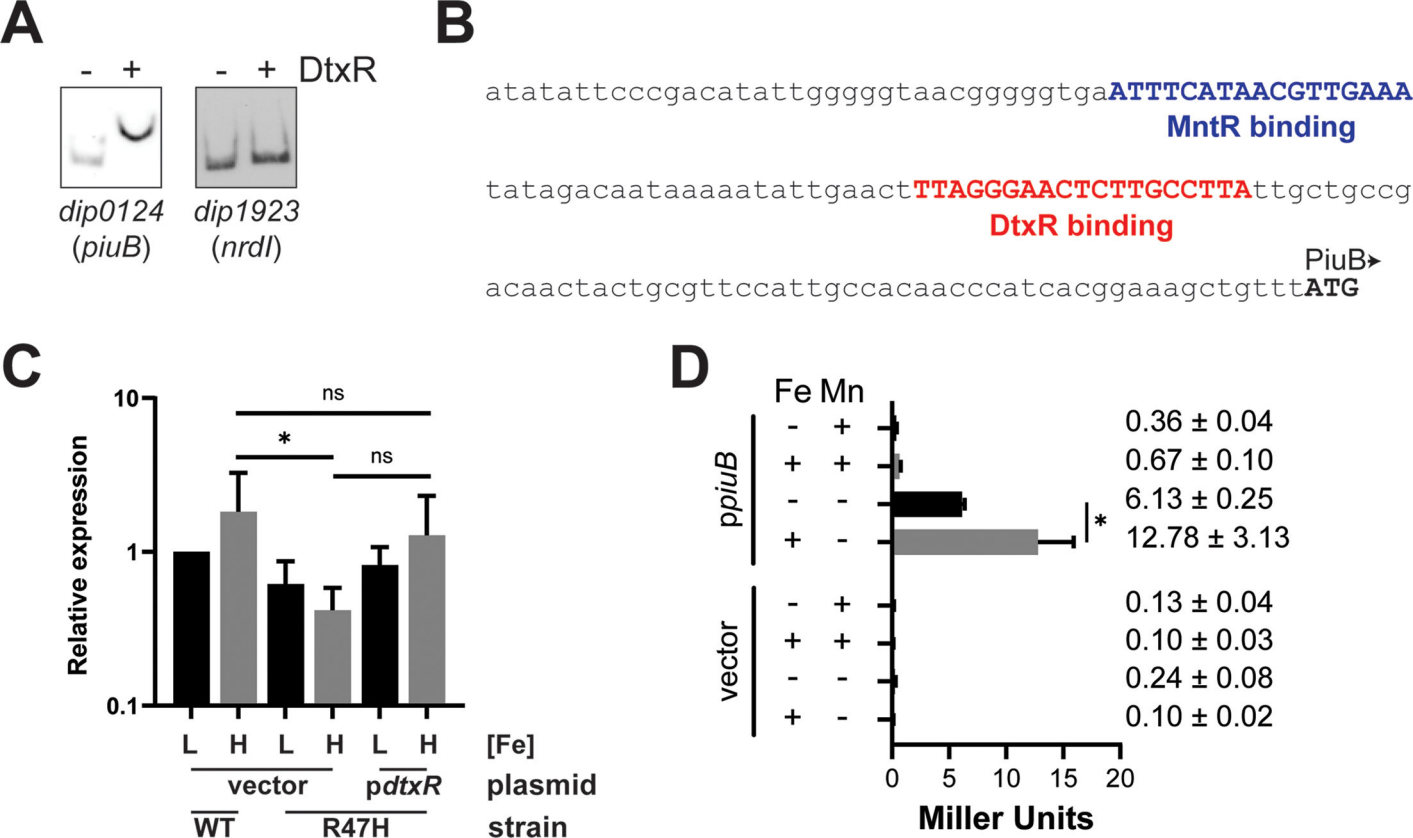
The *piuB* gene is regulated by iron and Mn. (**A**) EMSA results with biotinylated DNA target regions detected in the absence (−) or presence (+) of purified DtxR using the promoter region of *piuB* (*dip0124*) and negative control gene *nrdI* (*dip1923*). (**B**) The promoter region of *piuB* with putative MntR and DtxR-binding sites is indicated. (**C**) qPCR analysis of *piuB* expression from the indicated strain/plasmids grown in either low- (L, 0.25 µM) or high- (H, 5 µM) iron conditions. R47H indicates the *dtxR^-^* strain. Graphs show means with standard deviations of at least three replicates. Unpaired t tests were performed on indicated comparisons: *P <0.05 and ns for not significant. (**D**) LacZ activity was measured from cultures of *C. diphtheriae* 1737 WT carrying empty pSPZ vector (vector) or the promoter probe plasmid pSPZ-piuB (p*piuB*). Strains were grown in mPGT medium with high (+; 5 µM) or low (−; 0.25 µM) iron and with (+; 5 µM) or without (−) manganese supplementation. *P <0.05 by unpaired t test.

Among the Mn-regulated genes, only *piuB* is iron regulated in the wt strain (Table 3, wt, H:L), suggesting that DtxR may have a direct role in *piuB* expression. Although the function of the *piuB* gene product in *C. diphtheriae* is not known, the PiuB protein contains a PepSY domain that is known to have a diverse range of activities in bacteria, including siderophore iron utilization and peptidase regulation (38). We previously showed using an electrophoretic mobility shift assay (EMSA) that the Mn regulator, MntR, binds upstream of the start codon for the *piuB* gene (36). We show in Fig. 2A that DtxR also binds in the upstream region of *piuB*, suggesting that DtxR is directly involved in the iron-dependent regulation of *piuB* transcription. The putative binding sites for MntR and DtxR in the region upstream of the *piuB* gene are shown in Fig. 2B, and their location is based on the consensus binding sites for these transcriptional regulators. Since the transcription start site for the *piuB* gene could not be identified in a previous study (28), the location of the promoter elements for *piuB* is not known, and it is unclear how the binding of DtxR and MntR at their respective binding sites impacts gene expression. A control for the EMSA showed that DtxR does not bind in the upstream region for the MntR-regulated gene *nrd1* (*dip1923*), as expected (Fig. 2A). To better understand the regulation of the *piuB* gene, we measured the expression of *piuB* using both qPCR and a *piuB*-LacZ promoter fusion. Although the qPCR results in general support the finding from the RNA-seq data that the cloned wt copy of the *dtxR* gene is able to complement the *dtxR* mutation (Fig. 2C), a statistically significant difference in the expression of *piuB* in high-iron conditions was not established between the mutant carrying the vector and the mutant carrying the cloned *dtxR* gene due to the variability of the assay. However, the data showed a consistent trend supporting restoration of the wt phenotype for the mutant. In further support of the complementation of the *dtxR* mutant by the cloned *dtxR* gene, no significant difference in expression in high-iron conditions was observed between the wt strain carrying only the vector and the *dtxR*-complemented mutant (Fig. 2C). The LacZ results showed that expression from the *piuB* promoter was repressed by high levels of Mn (5 μM) as observed previously (36), and the Mn repression of *piuB* was maintained even in the presence of 5 μM iron (Fig. 2D). In the absence of both Mn and iron, 6.13 units of activity was detected, which was increased two-fold with the addition of 5 μM iron (12.78 units); consistent with the iron-dependent induction of *piuB* gene expression that was observed in the RNA-seq results. Minimal LacZ activity was observed in the vector control. The findings suggest that the regulation of *piuB* expression is complex and that it is the only *C. diphtheriae* gene known to be regulated by both DtxR/iron and MntR/Mn.

### Genes that displayed atypical regulation by iron and DtxR

We also identified numerous genes from the RNA-seq analysis whose expression and regulation by iron and DtxR differed from those in Tables 1 and 2. In Table S1, we show the RNA-seq results for 17 genes observed to be only regulated by iron in the wt strain. The most notable gene in this group was *dip0281* (*cat*), which encodes the catalase enzyme, a protein critical for protection from oxidative damage that may occur during growth in high-iron environments. The *cat* gene was induced in high-iron conditions in the wt strain and also in the *dtxR* mutant. It is possible that the iron regulation of some of the genes in Table S1, including *cat*, may be dependent on DtxR, but the difference in expression between wt and the *dtxR* mutant was below the threshold for statistical significance in the RNA-seq analysis.

In Table S2, RNA-seq results are shown for 83 genes that exhibited differential regulation between wt and the R47H *dtxR* mutant in high-iron conditions (R47H:wt, H:H). The genes in Table S2 did not exhibit differential regulation by iron levels in the wt strain. Interestingly, many of the genes in Table S2 (65 genes) were also regulated by iron in the *dtxR* mutant (R47H, H:L), and most of these genes showed elevated expression levels in the high-iron medium in the *dtxR* mutant, conditions that are expected to have high intracellular iron levels. These results indicate that the elevated expression of these genes in high iron in the *dtxR* mutant is independent of DtxR and is likely due to other regulatory proteins responding to stress conditions associated with growth in high intracellular iron environments. In support of this possibility, numerous genes encoded by bacteriophage β on the *C. diphtheriae* chromosome (*dip0180-dip0222*) are induced under these high-iron conditions (Table S2). The induction of these phage genes may be due to an iron stress response, which was observed for phage genes in other *Corynebacterium* species (39). Interestingly, the *tox* gene (*dip0222*) encoded on phage β is not induced by high iron (as are many of the other phage genes), but is repressed by iron and DtxR (Table 1) (3). It was also noted that numerous genes that showed elevated expression under high-iron conditions only in the *dtxR* mutant are predicted to express proteins that are associated with stress conditions, many of which have putative roles in DNA damage repair, including: *dip0134*, *dip0135*, *dip0268*, *dip0589*, *dip0604*, *dip0612*, *dip0722*-0*723*, *dip1026, dip1182*, *dip01450*, *dip1861, dip2023*, *dip2025*, *dip2132*, *dip2304*, and *dip2372*.

Only genes with the greatest differential expression are shown in Table S2, and the complete list of genes with this mode of regulation is provided in Table S3.

### Identification of new DtxR-binding sites

**Fig 3:**
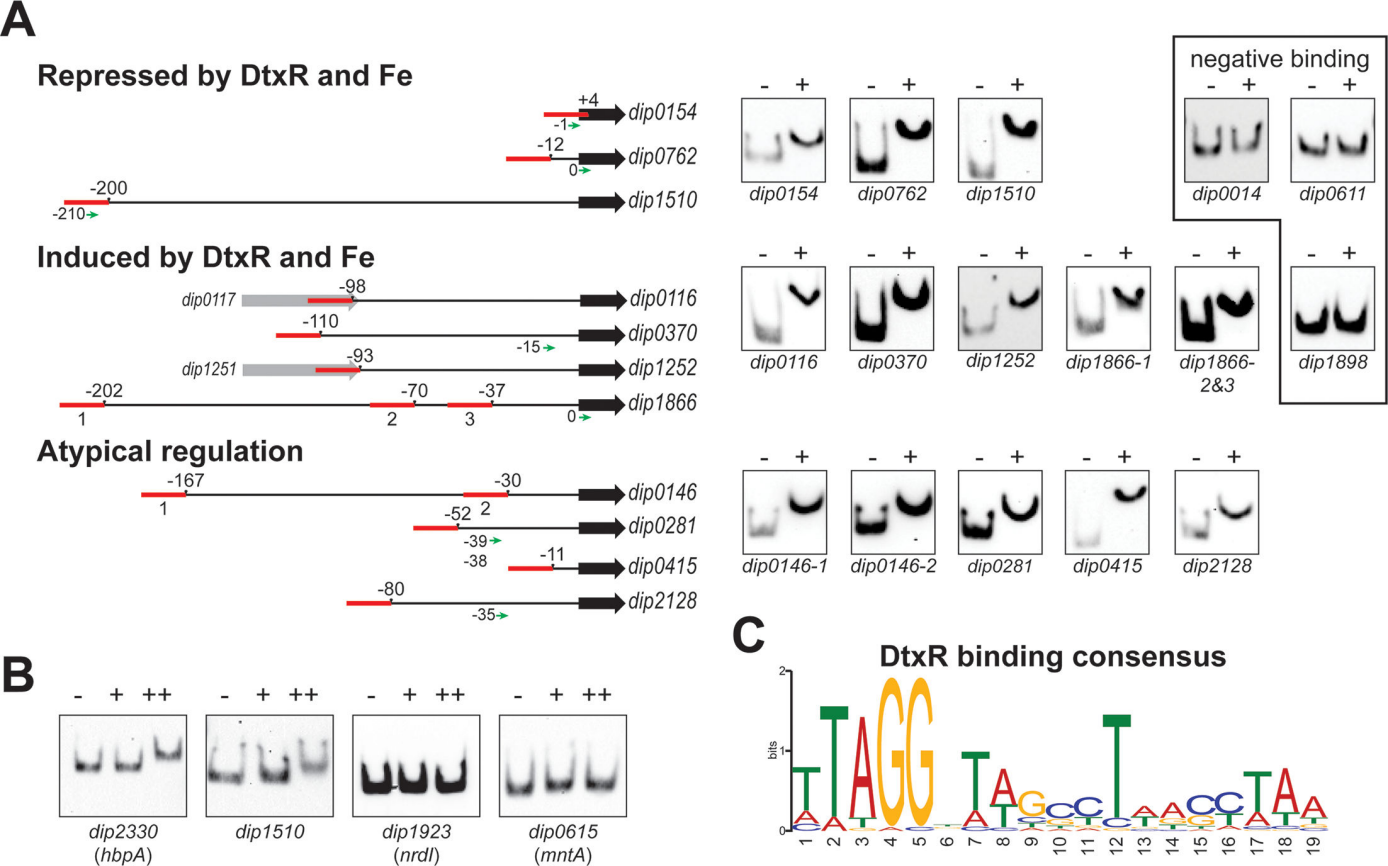
DtxR binding to the promoter region of novel iron- and DtxR-regulated genes was assessed using EMSAs. (**A**) Positions of putative DtxR-binding sites (red boxes) relative to the coding region for the indicated genes. The transcriptional regulation of the various genes examined is categorized as repressed by DtxR and iron, induced by DtxR and iron, or atypical regulation. Corresponding EMSA blots with biotinylated DNA target regions detected in the absence (−) or presence (+) of purified DtxR for each are shown (right). A subset of genes that fall within the gene regulation category was shown to have no binding to DtxR (negative binding inset). (**B**) Analysis of apo-DtxR and Fe-DtxR binding to selected promoter regions: (−) no DtxR or iron, (+) DtxR added, (++) DtxR and iron added. (**C**) DtxR-binding consensus sequence generated from verified DtxR-binding sites validated using the MEME suite (40).

Our analysis identified numerous genes for which DtxR and iron-dependent regulation were not previously described. We used the MEME suite algorithm (40) to assist in the identification of putative DtxR-binding sites upstream of several of the newly identified DtxR and/or iron-regulated genes (Fig. 3A). We then performed EMSAs to determine whether purified DtxR could bind to DNA fragments carrying the predicted sites indicated in Fig. 3A. All of the upstream regions that had predicted DtxR-binding sites bound DtxR, suggesting that DtxR is directly involved in the transcriptional regulation of these target promoters (Fig. 3A). DtxR binding was verified not only for genes that displayed iron-dependent expression by DtxR but also for genes that showed atypical expression by either iron or DtxR. Interestingly, the upstream region for gene *dip0014*, which was strongly repressed by iron in a DtxR-dependent manner (Table 1), did not bind DtxR in the EMSA (Fig. 3A). Additionally, *dip0611*, which is regulated by iron and DtxR, also failed to bind DtxR in its upstream region; however, the expression of *dip0611* was also regulated by iron in the *dtxR* mutant (Table 1), which suggests that factors other than DtxR are likely involved in the regulation of *dip0611*. The mechanism as to how *dip0014* is repressed by iron and DtxR has not been determined, but the findings suggest that DtxR has an indirect role in *dip0014* regulation.

Additionally, we tested the binding of apo-DtxR to promoter regions using purified DtxR dialyzed against metal-chelating agents (Fig. 3B). While binding was not observed under any of the conditions in the negative controls, which included two Mn- and MntR-regulated genes (*dip1923* and *dip0615*), our positive controls, *dip2330* and *dip1510*, showed binding to DtxR only with the addition of iron (Fig. 3B). Based on the DtxR-binding sites identified from the current study and from previous reports, a new DtxR consensus sequence was generated (Fig. 3C).

### DtxR directly induces *C. diphtheriae* ferritin expression

**Fig 4:**
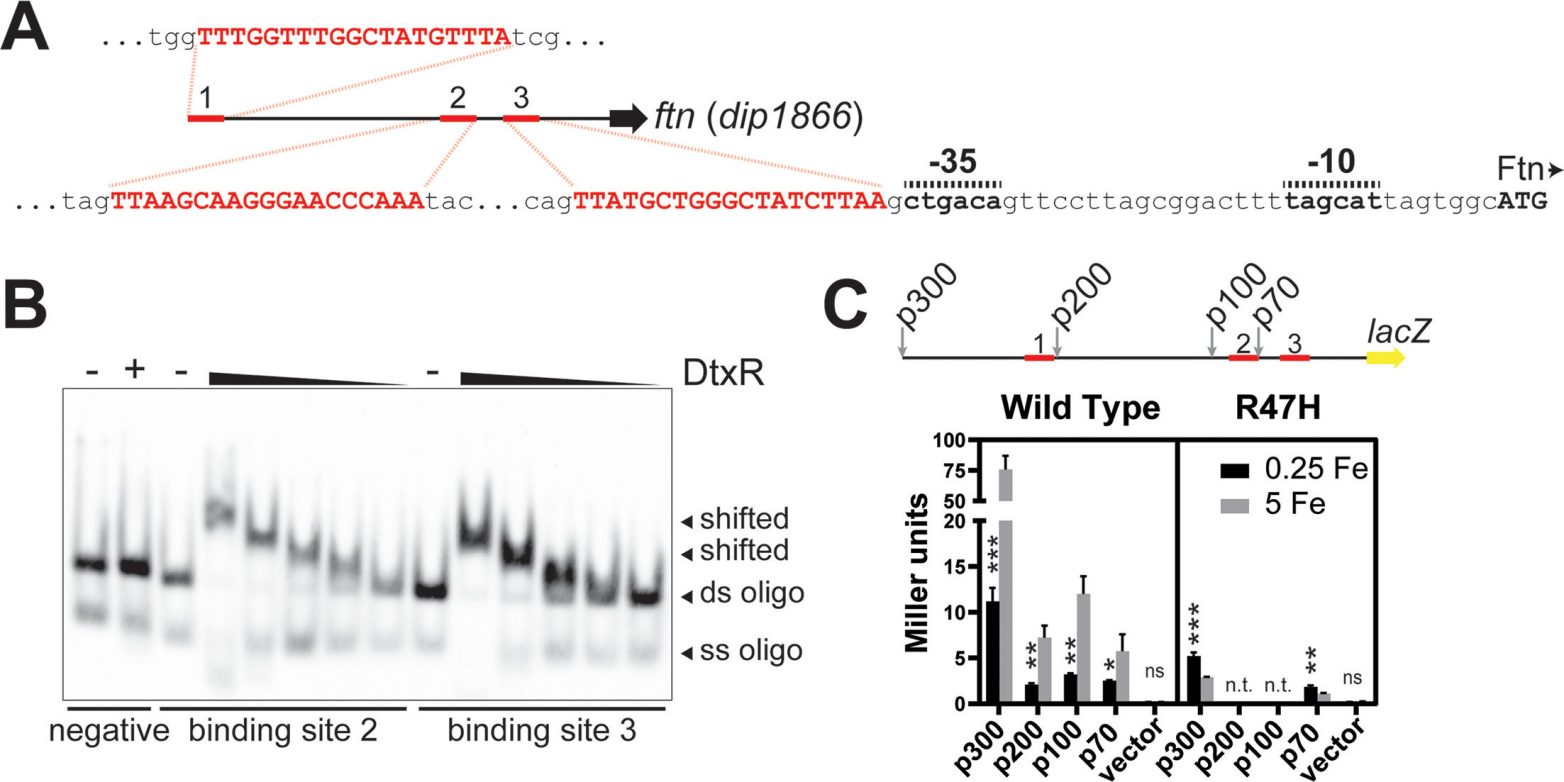
DtxR directly induces transcription of the *ftn* gene in high-iron conditions. (**A**) Sequences of the three DtxR-binding sites found upstream of the *ftn* gene (*dip1866*) with the transcriptional start site (Ftn) and promoter elements indicated. (**B**). EMSAs were performed using annealed oligonucleotides spanning the indicated 19-base putative binding motif and three adjacent bases. A negative control sequence was run alongside binding sites 2 and 3. DtxR was added where indicated (+), and serial twofold dilutions of DtxR were tested with the two binding sites. (**C**) LacZ activity was measured from cultures of *C. diphtheriae* 1737 WT and R47H (*dtxR*^-^) carrying pSPZ (vector) or the promoter probe plasmids carrying various regions of the *ftn* promoter region. The line diagram shows promoter fragments relative to identified binding sites. Strains were grown in mPGT medium with the indicated levels of iron supplementation (µM); n.t., not tested. Unpaired t tests were used to compare results for each plasmid grown in 0.25 Fe and 5 Fe: *** *P* <0.001; **P <0.01; *P <0.05; and ns for not significant.

The *ftn* gene (*dip1866*), which encodes a putative non-heme ferritin, was identified as one of the iron- and DtxR-activated genes (Table 2). In *C. diphtheriae*, direct activation of gene expression by DtxR is not well understood; however, the presence of three DtxR-binding sites upstream of the promoter region (Fig. 4A) as well as induction by iron suggests a direct role for DtxR in the regulation of *ftn* transcription. DtxR binding was verified upstream of the *ftn* gene using a DNA fragment harboring binding site 1 (*dip1866-1*; Fig. 3A), and a separate DNA fragment carrying both sites 2 and 3 (*dip1866-2&3*; Fig. 3A). Using biotinylated oligonucleotides for the two downstream sites, we confirmed that DtxR binds independently at both binding sites 2 and 3 (Fig. 4B). The −35, −10, and transcriptional start site of *ftn* were previously reported (28) and are indicated in Fig. 4A. Interestingly, the −35 is positioned one base downstream from DtxR-binding site 3, and the *ftn* transcript is predicted to lack a 5′ untranslated region (UTR) (28). The requirement for DtxR in the expression of the *ftn* gene was further confirmed in Western blots that showed that Ftn was strongly detected in the wt strain only under high-iron conditions and that Ftn was not detected in the R47H mutant (*dtxR*^-^) regardless of the iron levels, indicating a requirement for DtxR in the expression of *ftn* (Fig. 1C).

To determine the role of the various DtxR-binding sites in the iron- and DtxR-dependent induction of *ftn* gene expression, we designed a series of promoter-LacZ fusions that contained varying amounts of the *ftn* upstream region (Fig. 4C). The fusions were designed such that all sequence upstream of the start codon for the *lacZ* gene was derived from the *C. diphtheriae ftn* upstream region with no intervening plasmid sequence; this construction is predicted to mimic the configuration of the native *ftn* gene to result in a *lacZ* transcript lacking a 5′ UTR. We measured ß-galactosidase activity following growth in low- and high-iron conditions and observed that all promoter fusion constructs for the wt strain exhibited increased LacZ activity following growth in high iron, consistent with increased transcripts detected in the RNA-seq analysis. The construct with the largest amount of upstream region, p300, harbors all three DtxR-binding sites and resulted in the highest levels of activity in both low- and high-iron conditions. The p200 construct, which removes DtxR-binding site 1, resulted in reduced overall activity following growth in low- and high-iron conditions, but stimulation by iron was still observed. Similar levels of activity were observed from the p100 construct, which possesses the same number of DtxR-binding sites as p200, suggesting that the removal of the region between binding sites 1 and 2 has minimal effect on promoter activity. The p70 construct, which contains only DtxR binding site 1, resulted in the weakest overall β-galactosidase activity, but activity was still increased under high-iron conditions, indicating that the DtxR-binding site closest to the coding region is sufficient for induction by iron. To test whether DtxR is required for the increased expression in response to iron, the p300 and p70 fusions were introduced into the R47H mutant (*dtxR*^-^). Activity from both p300 and p70 in the R47H mutant was significantly weaker than activity detected in the wt strain, with the p300 fusion showing stronger activity than the p70 fusion (Fig. 4C). Furthermore, induction by iron was abolished for both constructs in the R47H mutant. Together, our results suggest a mechanism in which DtxR binding at the *ftn* promoter region is required for iron-responsive induction of *ftn* expression, and the DtxR-binding site adjacent to the −35 element (binding site 3) is sufficient for DtxR-dependent activation.

### RipA-regulated genes

**Fig 5:**
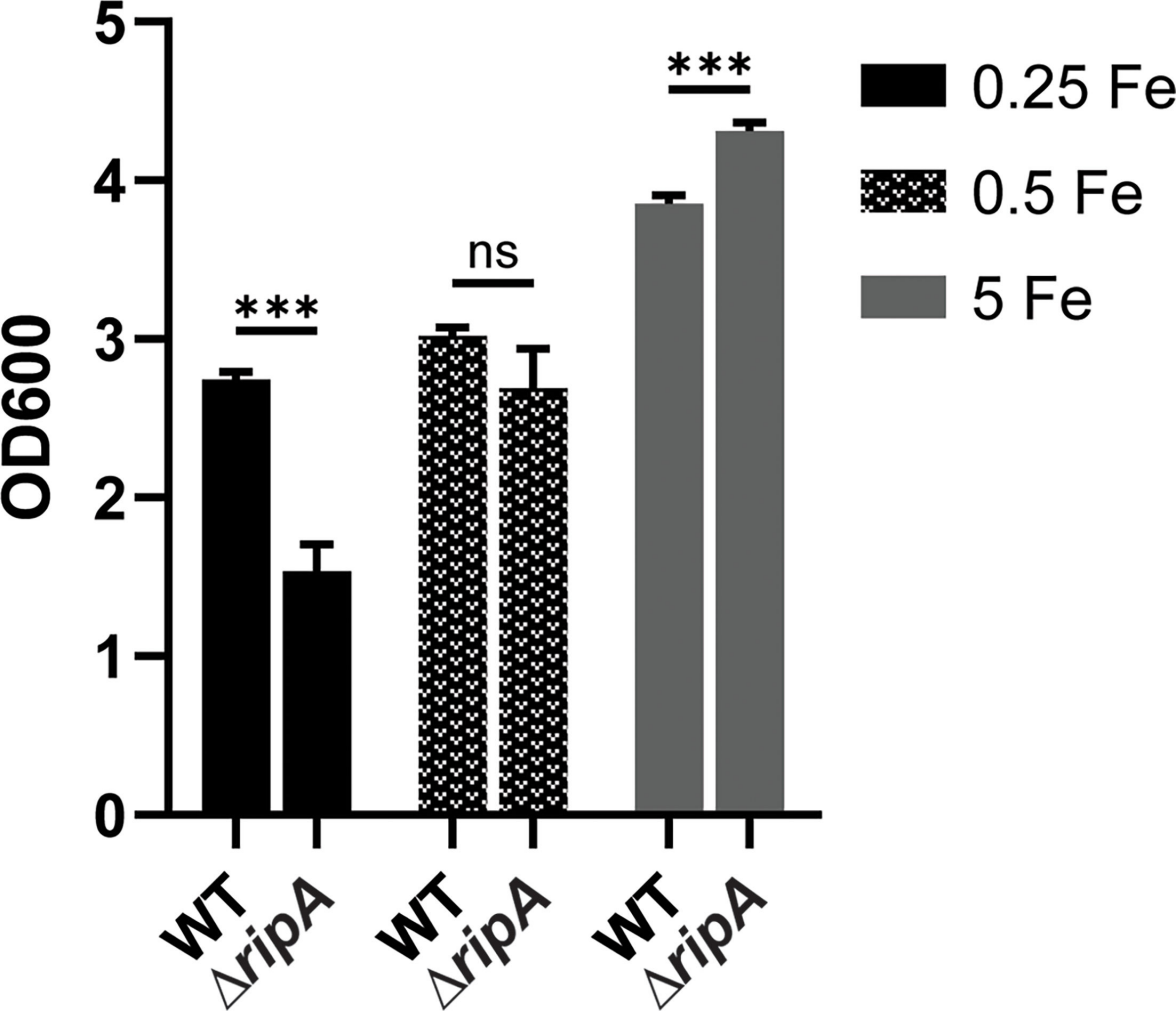
Growth of wt *C. diphtheriae* strain 1737 and the 1737Δ*ripA* mutant was assessed in low (0.25 µM and 0.5 µM) and high-iron (5 µM FeCl_3_) mPGT media. Results show the mean and standard deviation from at least three experiments. ***P <0.001 and ns for not significant by unpaired t test.

The expression of genes regulated by the RipA repressor protein is predicted to be optimally transcribed under high-iron conditions, since expression of the *ripA* gene is repressed by DtxR in high-iron media. Based on this mode of regulation, it is predicted that RipA-regulated genes should be fully expressed regardless of the iron levels in a *ripA* mutant. In addition to the RipA-regulated *acn* gene, which encodes aconitase, it is possible that additional genes in *C. diphtheriae* whose expression is elevated in high-iron conditions in a DtxR-dependent manner are also regulated by RipA (Table 2). In *C. glutamicum*, several genes were shown to be RipA repressed, which included the *acn* gene as well as genes that encode proteins for the production of succinate dehydrogenase and nitrate reductase (33). In *C. diphtheriae*, expression of genes encoding the succinate dehydrogenase complex (*dip0370-dip0372*), the nitrate reductase system (*dip0497-dip0501*) as well as aconitase (*acn*, *dip1283*) was shown to be induced by iron and DtxR in the RNA-seq study (Table 2). To determine how the expression of these genes, as well as three other similarly iron- and DtxR-induced genes, is affected in a *ripA* mutant, we examined the expression of these genes using promoter-*lacZ* fusions in the wt strain and in a *ripA* deletion mutant grown under high- and low-iron conditions. The *hbpA* gene, in which expression is iron and DtxR repressed and is not expected to be regulated by RipA, was used as a control for this study. Prior to initiating these studies, we noted that the growth of the *C. diphtheriae ripA* deletion mutant was reduced under low-iron conditions compared to wt (0.25 μM FeCl_3_), but had similar growth to the wt strain in high-iron conditions (Fig. 5); similar growth characteristics were previously reported for a *C. glutamicum ripA* mutant (33).

**Fig 6:**
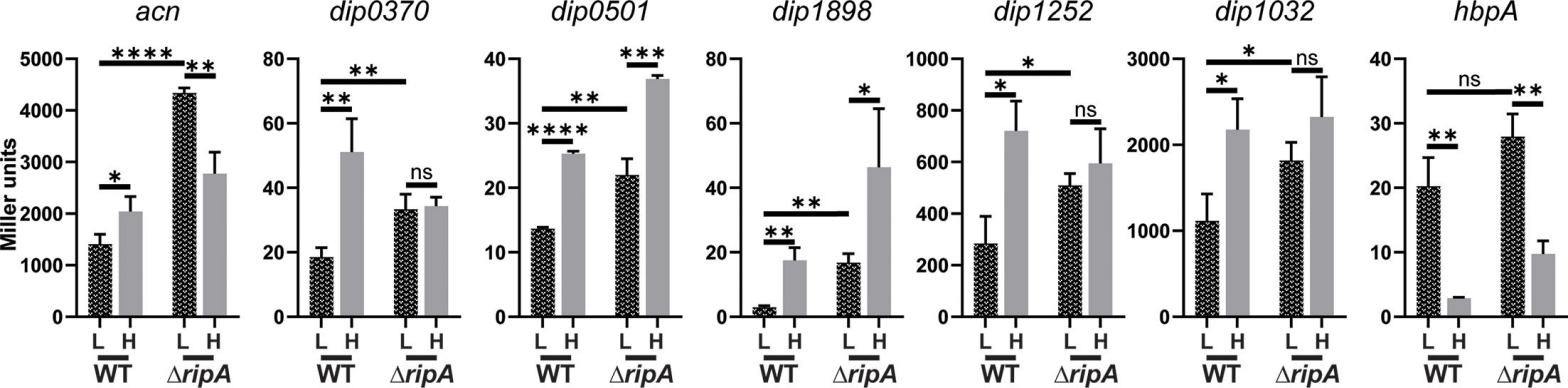
LacZ activity was measured from cultures of *C. diphtheriae* 1737 WT and 1737Δ*ripA* carrying the pSPZ plasmid with the promoter region indicated above each graph. Strains were grown in mPGT medium with high (H; 5 µM) or low (L; 0.5 µM) iron. The empty vector control had <0.5 units of activity under all of the conditions examined. ****P <0.0001; ***P <0.001; **P <0.01; *P <0.05; and ns for not significant by unpaired t test.

The results of the promoter-*lacZ* analysis showed that the expression of all of the genes that were induced in high iron in the RNA-seq analysis also exhibited significant increases in expression in high iron in the LacZ study (Fig. 6); this finding validates the use of the LacZ reporter for assessing the expression of this group of iron-regulated genes. All of the iron-induced genes also showed significantly increased levels of expression in the *ripA* deletion mutant in the low-iron medium compared to expression levels in the wt strain in the low-iron medium (Fig. 6). This result is consistent with the expression levels that would be expected in a *ripA* deletion mutant for a RipA-regulated gene. In the absence of the RipA protein, RipA-repressed genes should show elevated expression levels relative to their expression in the wt strain. As expected, expression of the *hbpA* control showed similar levels of iron-dependent repression in both wt and the *ripA* mutant, and no significant difference in expression in low-iron conditions between wt and the *ripA* mutant was observed for *hbpA* (Fig. 6). It was further observed that three of the genes (*acn*, *dip0501*, and *dip1898*) showed differences in expression between high- and low-iron conditions in the *ripA* mutant (Fig. 6). Differences in expression between high- and low-iron conditions in the *ripA* mutant were also observed previously for the *C. glutamicum acn* gene (33), which may be associated with the additional regulators that are known to control the expression of *acn* in *C. glutamicum* (41). It is also possible that additional regulators impact the expression of RipA-regulated genes in *C. diphtheriae*, but additional studies will be required to confirm this possibility.

## DISCUSSION

In the current study, we observed that expression of the ferritin gene, *ftn*, was strongly induced by iron and DtxR. Understanding how expression of the *ftn* gene is regulated by iron is of significant interest since ferritins are known to contribute to the virulence of various bacterial pathogens (42, 43). Because of the unusual DtxR- and iron regulation of the *ftn* gene, we explored the mechanism of regulation for this gene in greater detail. We found that *ftn* is directly induced by DtxR in the presence of iron, with DtxR binding directly to three independent sites upstream of the *ftn* promoter region. Transcriptional regulation of the *ftn* gene in *C. diphtheriae* shares some similarities to the regulation of the *Mycobacterium tuberculosis* ferritin gene, *bfrB*. The expression of *bfrB* was shown to be controlled by two separate factors, the Lsr2 repressor, an H-NS-like protein that is involved in both gene expression and chromosome structure, and IdeR, an ortholog of DtxR (44). The *bfrB* promoter contains two Lsr2-binding sites and four IdeR-binding sites configured in two groups of two adjacent binding sites. One of the Lsr2 sites is located between the two groups of IdeR-binding sites, while the second Lsr2 site is downstream of the *bfrB* promoter. Like DtxR, IdeR responds to intracellular iron levels. The authors proposed that binding of IdeR displaces Lsr2 near the IdeR-binding sites and alleviates Lsr2 repression to allow for transcription by the *bfrB* promoter in iron-replete conditions. *C. diphtheriae* encodes an Lsr2 homolog (*dip2266*); however, a role for *dip2266* in *ftn* expression has not been examined. It is possible that DtxR binding at site 3, which is adjacent to the *ftn* −35 promoter element, may facilitate RNA polymerase recognition of the promoter region and result in enhanced transcription of the *ftn* gene. Additional studies are needed to determine if DtxR interacts with an Lsr2 homolog or another DNA-binding protein at the *ftn* promoter in *C. diphtheriae*.

Prior to this report, only the gene encoding aconitase (*acn*) was shown to be RipA regulated in C. *diphtheriae* (34). In this study, we found several genes induced by iron in the RNA-seq analysis to be homologous to genes that were RipA regulated in *C. glutamicum* (33). A transcriptomic analysis of the RipA regulon in *C. glutamicum* identified 13 genes that were repressed by RipA in a low-iron medium (33). We searched for *C. diphtheriae* orthologs to these 13 *C*. *glutamicum* RipA-regulated genes (33) and assessed their expression profile in the RNA-seq analysis. Among the 13 orthologous genes, the *C. diphtheriae sdhCAB* (*dip0370-0372*), *acn* (aconitase, *dip1283*), *pta* (*dip2055*), and the *nar* operon genes (*dip0501-0497*) were found to be iron- and DtxR-induced, whereas the *leuCD* (*dip1127-1128*) genes did not show significant iron regulation. The *cat* gene (*dip0281*) is known to be iron and DtxR induced and RipA regulated in *C. glutamicum* (33), but was found to be only iron regulated in *C. diphtheriae*. However, the upstream region for the *cat* gene contains a DtxR-binding site consistent with the DtxR binding observed in the EMSA (Fig. 3A), suggesting that DtxR has a role in the regulation of the *cat* gene. Many of the genes regulated by RipA in *C. glutamicum* encode iron-containing enzymes involved in basic metabolism, and it is likely that other regulators affect the expression of these genes; for example, the expression of the *C. glutamicum acn* gene is regulated by at least four transcriptional regulators in addition to RipA (45). Our previous study of *C. diphtheriae* RipA revealed that expression of the RipA-regulated aconitase gene is complex and likely controlled by factors in addition to RipA (34), which may explain some of the unexpected expression profiles observed for a few of the putative RipA-regulated genes examined in this study (Fig. 6).

While our study focused primarily on genes found to be both iron- and DtxR-regulated, we identified many genes that appear to be differentially regulated only by iron (Table S1) or DtxR (Table S2). Many of the genes regulated only by DtxR encode proteins that are typically associated with DNA repair and are often produced during periods of oxidative stress, as might be expected under high-iron conditions. Many of the proteins in Table S2 were induced in the *dtxR* mutant under high-iron conditions, an environment that is expected to result in high intracellular iron levels. Most of the iron uptake systems in *C. diphtheriae* are repressed by DtxR in high-iron conditions in the wt strain (Table 1), and are, therefore, strongly expressed in the *dtxR* mutant, where these uptake systems would likely transport iron into the cell under conditions where intracellular iron levels are already high. While it is not known whether any of the genes induced in the *dtxR* mutant in high-iron conditions, such as *dip0611*, are directly controlled by DtxR, a DtxR-binding site was not found in the *dip0611* upstream region (Fig. 3A), which suggests that the intracellular environment in the *dtxR* mutant may result in the activation of specific regulatory factors that respond to these high-iron stress conditions.

Among the genes that were only differentially regulated by iron in the R47H mutant (Table S2) were numerous genes with putative roles in oxidative stress. Since DtxR mediates intracellular iron homeostasis in *C. diphtheriae*, the R47H mutant likely experiences some level of oxidative stress due to its inability to repress genes involved in iron uptake. The 1737 R47H mutant showed reduced growth in the high-iron medium, consistent with stress conditions likely due to elevated intracellular iron levels (Fig. 1A).

Together, the results from this study identified new roles and targets for *C. diphtheriae* DtxR regulation. We have characterized DtxR binding at newly identified iron-regulated promoters, with several of these promoters exhibiting non-canonical iron and DtxR regulation.

## MATERIALS AND METHODS

### Strains, media, and growth conditions

*C. diphtheriae* strain 1737 (46) and mutant derivatives (Table S4) were routinely cultured in heart infusion broth with 0.2% (vol/vol) Tween 80 (HIBTW) or on heart infusion agar (HIA; 1.5% agar) at 37°C. Strains were stored at −80°C in heart infusion broth with 20% (vol/vol) glycerol. Modified mPGT medium was prepared as described previously (47) except that increased amounts of Chelex100-treated Casamino acids were added (1.5% instead of 0.5%) to support growth of the R47H strain. Low- and high-iron conditions are defined as mPGT containing 0.5 and 5 µM FeCl_3_, respectively.

Kanamycin was used at 25 µg/mL and 50 µg/mL for *C. diphtheriae* and *E. coli*, respectively. Spectinomycin was used at 100 µg/mL for *C. diphtheriae* and *E. coli* strains. For experiments with the R47H strain, strains were grown on HIA supplemented with 5µg/mL ethylenediamine-N,N′-diacetic acid (EDDA); instead of HIBTW, strains were grown in mPGT with 0.25 and 5 µM FeCl_3_ used for low- and high-iron conditions, respectively, and stored in mPGT, 0.5 µM FeCl_3_ and 20% (vol/vol) glycerol at −80°C.

### Growth assays** **

Colonies from HIA plates with appropriate additives were used to inoculate HIBTW or mPGT cultures. Cultures were incubated overnight at 37°C with shaking, then diluted with fresh media (1:1). The diluted cultures were grown for an additional 4–6 h for mPGT cultures or 1–2 h for HIBTW cultures. For HIBTW cultures, after this incubation, 500 µL of culture was harvested and the cells were pelleted, resuspended in mPGT supplemented with 1 µM FeCl_3_, and grown for an additional 3–5 h. The cultures were then used to inoculate 1 mL mPGT at an OD_600_ of 0.03 and grown overnight with shaking at 37°C; the growth measured at OD_600_ 16–20 h after inoculation is reported.

### DNA cloning and generation of *C. diphtheriae* mutants

Plasmids used in this study were generated using PCR amplification from wt *C. diphtheriae* strain 1737 genomic DNA and are listed in Table S4. The NEBuilder HiFi DNA assembly cloning kit (New England Biolabs, Inc.) was used to insert DNA into vectors pK18mobsacB (pKdtxRR47H), pSPZ (*lacZ* fusions), and pKN2.6z (*dtxR* complementation clones). Plasmid sequences were verified by DNA sequencing (Macrogen or Plasmidsaurus).

The *dtxRR47H* point mutation DNA fragment was constructed by Genscript and inserted into the pK18mobsacB plasmid as above. The mutation was then integrated into the *C. diphtheriae* 1737 genome using the same allelic exchange method described previously (48) with the addition of 5 µg/mL EDDA to HIA plates used for the sucrose selection stage of mutant construction due to the R47H point mutant’s intolerance of high-iron conditions. The point mutation was confirmed by PCR across the gene locus, followed by sequencing (Plasmidsaurus).

### RNA extraction

For RNA-seq and qPCR, *C. diphtheriae* strains were grown on HIA plates with 5 µg/mL EDDA and antibiotics as appropriate, at 37°C or 30°C. Single colonies were used to inoculate mPGT, 0.5 µM FeCl_3,_ and antibiotics as needed, and grown at 37°C with shaking overnight. Overnight cultures were then diluted to a final OD_600_ of 0.1 in mPGT with appropriate iron and antibiotic supplementation and grown to logarithmic phase. Iron was supplemented at 0.25 and 5 µM FeCl_3_. Cultures were harvested in logarithmic growth phase and resuspended in Zymo DNA/RNA Shield following centrifugation. Aliquots of this material were submitted for RNA-seq (SeqCenter), or RNA was isolated in-house for RT-qPCR. Cell lysis was done mechanically using Zymo ZR bashing bead lysis tubes and lysis buffer from the subsequently applied Zymo Quick RNA-miniprep kit. RNA integrity and concentration were assessed using an Agilent RNA 6000 Nano chip and Bioanalyzer.

### RNA sequencing

For RNA-seq, RNA extraction was conducted at SeqCenter using the ZymoBIOMICS Quick-RNA Miniprep Kit (Zymo Research, R1055) following the recommendations in the protocol. Final RNA concentrations were quantified using a Qubit, after which samples were treated with DNAse (RNAse free) (Invitrogen). Library preparation used Illumina’s Stranded Total RNA Prep Ligation with Ribo-Zero Plus kit and 10bp unique dual indices. SeqCenter obtained a custom rRNA depletion probe pool using *C. diphtheriae* strains 1737 (NC_002935) and C7ß (CP003210). Sequencing was done on a NovaSeq X Plus, producing paired-end 150 bp reads. Demultiplexing, quality control, and adapter trimming were performed using bcl-convert. Quality control and adapter trimming were also performed using bcl-convert, read mapping used HISAT2, and read quantification was performed using Subread’s featureCounts functionality. Mapping statistics and raw, quantified counts were provided. Normalization of read counts used edgeR’s Trimmed Mean of M values algorithm, and values were converted to counts per million. Differential expression analysis used edgeR’s glmQLFTest. Differentially expressed genes were defined as those with logFC (base 2) > 1 and *P* < 0.05; see Supplemental material for all differentially expressed genes.

RNA-seq raw data can be found at the NCBI SRA database: ID- PRJNA1336240.

### cDNA synthesis and qPCR

The ProtoScript II First-Strand cDNA synthesis kit (New England Biolabs, Inc.) was used to synthesize cDNA from 100 ng of total RNA. qPCR was performed using the ViiA7 (Thermo Fisher Scientific) with Luna Universal qPCR master mix (New England Biolabs, Inc.). Primers used for qPCR were designed using Primer3 (49) and are listed in Table S5. Data were analyzed using the ΔΔCq method and significance determined using unpaired t tests on ΔCq values. ΔCq values for *gyrB* were used for normalization. *GyrB* (*dip0005*) was not differentially expressed in our analysis.

### Electrophoretic mobility shift assays

EMSA experiments using DtxR were performed as described previously (34). Biotinylated DNA probes were generated through PCR amplification of *C. diphtheriae* strain 1737 genomic DNA with biotinylated primers (Integrated DNA Technologies). For minimal DtxR-binding sites, complementary oligonucleotides were annealed by mixing equimolar amounts and denaturation for 2 m at 98°C in a thermocycler. The temperature was reduced from 98°C to 4°C in 1-m intervals; annealed oligomers were stored at 4°C until use. Primers used to generate DNA probes for the EMSAs are described in Table S6. Biotinylated DNA was detected using the LightShift chemiluminescent EMSA kit (Thermo Scientific).

DtxR was purified using the two-plasmid system for temperature-inducible expression as described previously (34). For testing metal-dependent DNA-binding activity of DtxR, elution fractions containing DtxR were dialyzed sequentially against (i) PBS with 1 mM DTT and 1 mM EDTA for 2 h followed by (ii) PBS with 1 mM DTT and 2 g/L Chelex100 for 2 h, and lastly (iii) PBS with 1 mM DTT, 2 g/L Chelex100, and 15% glycerol for 12 h. For DtxR-binding assays, purified DtxR was incubated at room temperature with target DNA in DtxR-binding buffer (20 mM Na_2_HPO_4_, 50 mM NaCl, 2 mM DTT, 5 mM MgCl_2_, 0.2 µg/µL BSA, 0.05 µg/µL sonicated salmon sperm DNA, and 0.5 mM FeSO_4_ in 10% glycerol at pH 7.0). A reaction in which DtxR was omitted was prepared in parallel. Samples were separated by gel electrophoresis (5% acrylamide with 45 mM Tris-borate [0.5×TB]) and transferred onto nylon in 45 mM Tris-borate, 1 mM EDTA (0.5× TBE).

### Beta-galactosidase assays

Overnight cultures of *C. diphtheriae* with indicated plasmids were grown in mPGT and 0.5 µM FeCl_3_. Cultures were diluted 1:1 with fresh medium and grown for 4–6 h. Cultures were then diluted to a final OD_600_ of 0.03 in fresh medium with indicated metal supplementation. Following overnight growth, cells were pelleted and treated with 10 mg/mL lysozyme in PBS at 37°C for 30 min. Following lysozyme treatment, β-galactosidase (LacZ) activity assays were performed as described by Miller (50). Experiments were performed using cultures initiated from different colonies on different days for a total of three replicates per strain and plasmid.

### Generation of antisera against ferritin

The gene encoding ferritin (*ftn*, *dip1866*) was cloned into pET24 with an N-terminal Strep II tag to generate the plasmid pET-1866. The plasmid was transformed into *E. coli* BL21(DE3), and protein was purified using Strep-Tactin XT resin (IBA Lifesciences) following the manufacturer’s recommendations. Elution fractions containing the target protein were dialyzed against PBS and used to immunize guinea pigs for antisera production (Cocalico Biologicals, Inc.).

### Cell lysis, electrophoresis, and Western blotting

Cells used for gel electrophoresis and Western blotting were grown in mPGT with FeCl_3_ supplementation as indicated and normalized by OD_600_ at harvest. After centrifugation and harvest of supernatant, cell pellets were lysed using lysozyme and sodium lauroyl sarcosinate as previously described (24). Normalized quantities of cell lysate or supernatant were boiled for 10 min in Laemmli buffer and separated on 4%–15% denaturing precast TGX (Tris-Glycine eXtended) PAGE gels using tris-glycine-SDS running buffer (all reagents from Bio-Rad). Coomassie staining, transfer, and western blot procedures were done as previously described (24). Anti-ferritin primary antibody was used at 1:2,000; IutE primary antibody at 1:5,000; HbpA primary antibody at 1:10,000; and DT (toxin) primary antibody at 1:10,000.

### Statistical analyses

GraphPad Prism v10.6.0 was used for data analysis. Specific tests and *P* values are noted in the figure legends.

## Data Availability

The genome sequence of *C. diphtheriae* strain 1737 is available in GenBank under accession number PRJNA1335246. The sequence reads from RNA-seq are available under the Sequence Read Archive (SRA) accession number PRJNA1336240. All gene designations (DIP#) used throughout this study are based on the annotation of strain NCTC13129.

## SUPPLEMENTAL MATERIAL

Supplemental tables jb.00530-25-s0001.docx Tables S1, S2, S4, S5, and S6.

Table S3 jb.00530-25-s0002.xlsx Genes regulated only by DtxR.

ASM does not own the copyrights to Supplemental Material that may be linked to, or accessed through, an article. The authors have granted ASM a non-exclusive, world-wide license to publish the Supplemental Material files. Please contact the corresponding author directly for reuse.
