# Supplementary material for: DtxR is a global iron-dependent regulatory protein with multiple roles in the control of gene expression in Corynebacterium diphtheriae
Source: J Bacteriol. 2026 Feb 26;208(3):e00530-25. doi: 10.1128/jb.00530-25 (PMC13001254; doi:10.1128/jb.00530-25)
Supplement: Supplemental tables — Tables S1, S2, S4, S5, and S6. [file jb.00530-25-s0001.docx]

**Supplemental Material**

**Table S1. Genes only regulated by iron**

| **Locus** | **Gene Description** | **Log_2_ Fold Change ^a^** | |
| --- | --- | --- | --- |
|  |  | **wt**  **H:L** | **R47H**  **H:L** |
| DIP_RS11610 | Sulfate-transport domain-containing protein | 1.55 | 1.12 |
| DIP0066 | secreted protein-unknown function | 1.69 |  |
| DIP0281 | *cat* \| Catalase | 1.63 | 1.26 |
| DIP0659 | secreted protein-unknown function | -1.80 | -1.03 |
| DIP0920 | GppA phosphatase family | 1.54 |  |
| DIP0975 | RNA psuedourdine synthetase family | 1.23 |  |
| DIP1027 | *yceI*-domain family-Polyisoprenoid-binding protein | -3.16 |  |
| DIP1096 | *ilvD* \| dihydroxy-acid dehydratase | 1.87 | 1.21 |
| DIP1201 | SEC-C domain protein | -1.56 |  |
| DIP1202 | A/B hydrolase family | -1.30 |  |
| DIP1448 | methyl transferase | 1.41 | 1.92 |
| DIP1496 | methionine aminopeptidase | -1.42 |  |
| DIP1629 | cytochrome oxidase | 1.05 | 1.01 |
| DIP1976 | Lipoprotein--Ig fold domain | 1.91 | 1.29 |
| DIP2080 | Hydroxyacid dehydrogenase | -1.45 |  |
| DIP2128 | peptide transport protein | -1.38 |  |
| DIP2264 | RNAse H | 1.63 | 1.12 |

^a^ Log_2_ fold change indicated. Compares relative signal intensity between the wt or R47H strains grown in high- (H) or low- (L) iron as indicated. Negative value (blue) indicates increased transcripts detected in the second condition listed; positive value (yellow/orange) indicates increased transcripts detected in the first condition.

**Table S2. Genes differentially regulated only in the R47H strain**

| **Locus ^a^** | **Gene Description** | **Log_2_ Fold Change ^b^** | |
| --- | --- | --- | --- |
|  |  | **wt:R47H**  **H:H** | **R47H**  **H:L** |
| DIP0013 | Long Rib domain-containing protein | -2.10 | 1.30 |
| DIP0041 | Pseudogene | -2.05 |  |
| DIP0078 | Hypothetical protein | -2.14 |  |
| DIP_RS24540 | PhzF family phenazine biosynthesis protein | -2.57 | 1.35 |
| DIP_RS23110 | hypothetical protein | -3.09 | 1.54 |
| DIP_RS11930 | hypothetical protein | -2.08 |  |
| DIP0101 | *ccdA* \| cytochrome c biogenesis protein | -3.00 | 1.42 |
| DIP0123 | ROK family protein | -2.46 | 2.07 |
| DIP0134 | DNA-3-methyladenine glycosylase I | -3.31 | 2.06 |
| DIP0135 | SDR family NAD(P)-dependent oxidoreductase | -2.35 | 1.69 |
| DIP0142 | hypothetical protein | -2.58 | 1.39 |
| DIP0146 | SdpI family protein | -2.11 | 1.94 |
| DIP0180 | phage beta genes | 1.65 |  |
| DIP0181 |  | 1.91 | -1.14 |
| DIP0182 |  | -3.79 | 3.06 |
| DIP0183 |  | -2.56 | 1.92 |
| DIP0184 |  | -2.49 | 1.98 |
| DIP0185 |  | -1.36 |  |
| DIP_RS12335 |  | -1.46 |  |
| DIP0187 |  | -2.26 |  |
| DIP0189 |  | -1.32 | 1.55 |
| DIP0190 |  | -1.49 | 1.24 |
| DIP0200 |  | -2.01 | 1.37 |
| DIP_RS12395 |  | -2.19 | 2.15 |
| DIP0201 |  | -1.97 | 1.08 |
| DIP0203 |  | -1.15 | 1.14 |
| DIP0204 |  | -1.14 |  |
| DIP0206 |  | -1.52 | 1.02 |
| DIP0207 |  | -1.55 |  |
| DIP0208 |  | -1.92 | 1.05 |
| DIP0209 |  | -1.30 |  |
| DIP0210 |  | -1.21 |  |
| DIP0211A |  | -1.39 |  |
| DIP0214 |  | -1.18 | 1.18 |
| DIP0216 |  | -1.04 |  |
| DIP0218 |  | -1.74 |  |
| DIP0219 |  | -1.48 | 1.30 |
| DIP0220 | end of phage genes | -1.40 |  |
| DIP0240 | Ig-like domain-containing protein | -2.29 |  |
| DIP0268 | HNH endonuclease signature motif containing protein | -3.45 | 1.99 |
| DIP_RS23860 | Hypothetical protein | -3.09 | 1.84 |
| DIP0337 | site-specific integrase | -2.40 | 1.91 |
| DIP0339 | Hypothetical protein | -2.78 | 1.90 |
| DIP0343 | Hypothetical protein | 2.51 | -1.80 |
| DIP0354 | IS110 family transposase | -2.49 | 1.59 |
| DIP0415 | ArsR/SmtB family transcription factor | -2.39 | 1.12 |
| DIP0418 | 1,4-dihydroxy-2-naphthoate polyprenyltransferase | -2.83 | 1.70 |
| DIP0419 | HNH endonuclease signature motif containing protein | -2.67 | 1.18 |
| DIP0514 | ABC transporter permease | 2.18 | -2.31 |
| DIP0556 | type VII secretion protein EccC | -2.02 | 1.18 |
| DIP0589 | EamA family transporter | -2.25 | 1.29 |
| DIP0602 | Hypothetical protein | -2.73 | 1.45 |
| DIP0603 ↓ | Hypothetical protein | -2.66 | 1.85 |
| DIP0604 | Y-family DNA polymerase | -3.46 | 2.38 |
| DIP0612 ↓ | *dnaE2* \| error-prone DNA polymerase (dip0612-0614)^c^ | -2.56 | 2.02 |
| DIP0722 ↓ | ATP-dependent DNA helicase (dip0722-0726)^c^ | -2.32 | 1.57 |
| DIP0723 | ATP-dependent helicase | -1.99 | 1.40 |
| DIP1010 | Hypothetical protein | -4.10 | 2.38 |
| DIP1026 | AAA family ATPase | -1.88 | 1.76 |
| DIP_RS24085 | Hypothetical protein | 2.01 | -1.70 |
| DIP1153 | TIGR04053 family radical SAM/SPASM domain-containing protein | 2.72 | -1.38 |
| DIP1182 | DNA repair protein RecN | -1.84 | 1.45 |
| DIP1450 | recombinase RecA | -2.04 | 1.68 |
| DIP1733 | HNH endonuclease signature motif containing protein | -2.06 | 1.21 |
| DIP1735 | ABC transporter ATP-binding protein | -2.38 | 2.03 |
| DIP1758 | Hypothetical protein | -2.40 | 1.25 |
| DIP1806 | carbon starvation CstA family protein | 2.68 | -3.34 |
| DIP1861 | HNH endonuclease signature motif containing protein | -3.54 | 2.05 |
| DIP1944 | IS6-like element IS6100 family transposase | -2.30 | 1.12 |
| DIP1977 | DNA repair protein RadA | -2.74 | 1.75 |
| DIP2023 | Fic family protein | -2.13 | 1.25 |
| DIP2025 | RtcB family protein | -2.09 | 1.10 |
| DIP_RS23705 | Hypothetical protein | -2.57 | 1.53 |
| DIP2132 | Oxidoreductase | -4.98 | 2.79 |
| DIP2153 | HNH endonuclease | -2.13 |  |
| DIP2206 | ATP-binding cassette domain-containing protein | -2.12 |  |
| DIP2235 | glycerol kinase GlpK | 1.18 |  |
| DIP2236 | MIP/aquaporin family protein | 1.81 |  |
| DIP2237 ↑ | glycerol-3-phosphate dehydrogenase/oxidase | 2.19 |  |
| DIP2241 | GntR family transcriptional regulator | 1.90 |  |
| DIP2304 | Fpg/Nei family DNA glycosylase | -2.03 |  |
| DIP2372 | RNA polymerase sigma factor | -2.17 |  |

^a^ Boxed genes indicate operons; arrows indicate the first gene in the operon and direction of transcription.

^b^ Log_2_ fold change indicated. Compares relative signal intensity between the wt or R47H strains grown in high- (H) or low- (L) iron as indicated. Negative value (blue) indicates increased transcripts detected in the second condition listed; positive value (yellow/orange) indicates increased transcripts detected in the first condition.

^c^ Parenthesis indicate full operon where not all genes in operon showed significant regulation.

**Table S3. Genes Regulated only by DtxR. See excel Table**

**Table S4. Bacterial strains and plasmids used in this study.**

| **Strain or plasmid** | **Purpose** | **Source** |
| --- | --- | --- |
| *C. diphtheriae* strains |  |  |
| 1737 (WT) | Wild type, Gravis biotype, tox^+^ | (1) |
| R47H | Point mutation in *dtxR* in 1737 at amino acid location 47 | (2) |
| Δ*ripA* | In-frame deletion of *ripA* in 1737 | (3) |
|  |  |  |
| *E. coli* strains |  |  |
| DH5α | Cloning and protein expression | New England Biolabs |
| S17-1 λpir | Mating strain | (4) |
| BL21DE3 | Protein expression strain | New England Biolabs |
|  |  |  |
| Plasmids |  |  |
| pGP1-2 | Encodes temperature-inducible T7 RNA polymerase; Kan^R^ | (5) |
| pMS298 | Encodes *C. diphtheriae dtxR* under T7 control; Amp^R^ | (6) |
| pSPZ | *lacZ* reporter plasmid; Spc^R^ | (7) |
| pSPZ0370 | *lacZ* fusion; contains *dip0370 (sdhC)* promoter | This study |
| pSPZ0501 | *lacZ* fusion; contains *dip0501* promoter | This study |
| pSPZ1032 | *lacZ* fusion; contains *dip1032* promoter | This study |
| pSPZ1252 | *lacZ* fusion; contains *dip1252* promoter | This study |
| pSPZ1866-300 (p300) | *lacZ* fusion; contains 300 bases of *dip1866* promoter | This study |
| pSPZ1866-200 (p200) | *lacZ* fusion; contains 200 bases of *dip1866* promoter | This study |
| pSPZ1866-100 (p100) | *lacZ* fusion; contains 100 bases of *dip1866* promoter | This study |
| pSPZ1866-70 (p70) | *lacZ* fusion; contains 70 bases of *dip1866* promoter | This study |
| pSPZ1898 | *lacZ* fusion; contains *dip1898* promoter | This study |
| pSPZacn | *lacZ* fusion; contains aconitase (*acn, dip1283)* promoter | This study |
| pSPZhbpA | *lacZ* fusion; contains *hbpA (dip2330)* promoter | (2) |
| pSPZpiuB | *lacZ* fusion; contains *piuB (dip0124)* promoter | This study |
| pK18mobsacB | Suicide vector | (8) |
| pKdtxRR47H | Vector to introduce R47H point mutation in *dtxR* | This study |
| pKN2.6z | *C. diphtheriae* shuttle vector, Kn^R^ | (9) |
| p2.6dxtR | pKN2.6Z carrying the wt *dtxR* gene | (10) |

**Table S5. Primers used for qPCR.**

| **Gene** | **Primer sequence (5’-3’)** | **Amplicon size (bp)** |
| --- | --- | --- |
| *dip1024 (piuB)* | gtatctgagcaggcacaacg | 198 |
|  | ccactgctcaaagtccacac |  |
| *dip0173 (iutE)* | ACCACGTCCCAGTGATCTTC | 171 |
|  | ACTTCAGGGCACCGATGTAG |  |
| *dip0222* (*tox*) | GAACAGGCGAAAGCGTTAAG | 224 |
|  | TTTTTGATAGGGCCATGCTC |  |
| *dip0370 (sdhC)* | ctctacccactgcttccaca | 220 |
|  | ggaagatgatgaacgccagc |  |
| *dip0615 (mntA)* | GTTGTGGGAGGAATCGTCAT | 150 |
|  | GTCAGCGTCGATTTGTTTGA |  |
| *dip1149* | cagctttaacgggatctggc | 163 |
|  | ataatgactgccgcgttgac |  |
| *dip2330 (hbpA)* | CTCTCGGGTGGAGAACAAAG | 152 |
|  | CTGCCTTGGAGTTGAGGAAG |  |
| *dip1866 (ferr)* | CTGGATGAAGAAGCAGCACA | 184 |
|  | GCGGATCATGCCAGTAATCT |  |
| *dip0005 (gyrB)* | GGTCTGACCATTACGCTGGT | 166 |
|  | TCTTCTCGCGTTTCTTTGGT |  |

**Table S6. Primer pairs used for EMSA targets.**

| **Gene Locus** | **Amplicon Size** ^a^ | **Primer Sequence(5'-3')** ^b^ |
| --- | --- | --- |
| DIP0014 | 150 | **B-**CCCCCCGTAGAGCTTCCAGCCGCAG |
|  |  | CCCCCCTGCACCATGCACAAAACCCCA |
| DIP0116 | 100 | **B-**CCACGATCCGCGAGTACG |
|  |  | TAGCGAACAAGGATTGAAACCC |
| DIP0124 | 100 | **B-**ACGGGGGTGAATTTCATAACG |
|  |  | GCAATGGAACGCAGTAGTTGT |
| DIP0146-1 | 100 | **B-**GACCAACCTTGTTTAGACAGAAC |
|  |  | CCACGCCAAAAATAGGGCAG |
| DIP0146-2 | 108 | **B-**CACATCGGCTGTGCGTCG |
|  |  | GCTGCGGCGATTGGCATG |
| DIP0154 | 150 | **B-**CCCCCCTGGCAGCACAAGAGCAGTG |
|  |  | CCCCCCCTAGTTAGGTTATCCAACATTGGG |
| DIP0281 | 130 | **B-**TAGCACTCCACCTTTAGTTGTAG |
|  |  | GGCTGGTCACGATGTCCC |
| DIP0370 | 157 | **B-**CCCCCCCATGGCACCTCCAGTGTCG |
|  |  | GCCACCCCAGAAGCCGTT |
| DIP0415 | 100 | **B-**TTCCTGTGGAAGCTCGAGC |
|  |  | ATGAACTATATAGGCAAGGTTAAGC |
| DIP0611 | 200 | **B-**CCCCCCCACACATGTTCACATATACATGAATA |
|  |  | CCCCCCCATGATTTGTCTTTCTGTTATGAGG |
| DIP0615 | 105 | **B**-CAATACGCTGAACAGTTAATCTTG |
|  |  | CACCAACGATTTTACGCATGATAC |
| DIP0762 | 124 | **B-**CCATACCGAAATTTTAATAACTAAGG |
|  |  | TGCCACTCACCCTGTCTAC |
| DIP1252 | 150 | CCCCCCGCTGATGCCCCTCCGTGT |
|  |  | CCCCCCCATTCTGGAATTCAGCACAATACT |
| DIP1510 | 100 | **B-**CCCACTGGCATAGATGTTAAAAC |
|  |  | TCAAAAAGCTTTACTTAGGCTTGC |
| DIP1866-1 | 100 | **B-**CCAAAATGTGACTTTCGGCAC |
|  |  | GTGAGATATGCCACGTTTGCC |
| DIP1866-2&3 | 100 | **B-**CGCAAAGCAATTATTCGAGCTG |
|  |  | AAGGAACTGTCAGCTTAAGATAG |
| DIP1898 | 250 | CCCCCCAGCAGCTTTTTTAGAACAATCCCT |
|  |  | CCCCCCCATCAGAGGAGAACTTTCTGC |
| DIP1923 | 136 | **B-**CAAGCATTAATGTATGCGGTTTTC |
|  |  | CCTTGCGGTGTAGTTGCGG |
| DIP2128 | 100 | **B-**TCGCGGTTTCTCGAGGATG |
|  |  | CACAACATTTTCAATAACGTTTTCAC |
| DIP2330 | 198 | **B**-GAAAAATAGGCCCCTAACTGAC |
|  |  | GAGGGTGGTCAGGATATTCAAG |
| DIP1866-2 | 31 | **B-**CCCTAGTTAAGCAAGGGAACCCAAATACGGG |
|  |  | CCCGTATTTGGGTTCCCTTGCTTAACTAGGG |
| DIP1866-3 | 31 | **B-**CCCCAGTTATGCTGGGCTATCTTAAGCTGGG |
|  |  | CCCAGCTTAAGATAGCCCAGCATAACTGGGG |
| Nonspecific | 31 | **B-**CCCAAGCACGCAGACGATGACGACTTCATGGGG |
|  |  | CCCCATGAAGTCGTCATCGTCTGCGTGCTTGGG |

^a^ Indicated size in number of bases, including residues integrated into the primer ends to improve dsDNA stability.

^b^ **B-** denotes biotinylation of the primer for detection purposes.

**Supplemental References:**

1. Popovic T, Kombarova SY, Reeves MW, Nakao H, Mazurova IK, Wharton M, et al. Molecular epidemiology of diphtheria in Russia, 1985-1994. J Infect Dis. 1996;174(5):1064-72.

2. Lyman LR, Peng ED, Schmitt MP. The *Corynebacterium diphtheriae* iron-regulated surface protein HbpA is involved in the utilization of the Hemoglobin-Haptoglobin complex as an iron source. Journal of bacteriology. 2018.

3. Peng ED, Oram DM, Battistel MD, Lyman LR, Freedberg DI, Schmitt MP. Iron and Zinc Regulate Expression of a Putative ABC Metal Transporter in Corynebacterium diphtheriae. J Bacteriol. 2018;200(10).

4. Simon R, Priefer U, Puhler A. A broad host range mobilization system for in vivo genetic engineering: transposon mutagenesis in Gram negative bacteria. Nat Biotech. 1983;1(9):784-91.

5. Tabor S, Richardson CC. A bacteriophage T7 RNA polymerase/promoter system for controlled exclusive expression of specific genes. Proc Natl Acad Sci U S A. 1985;82(4):1074-8.

6. Schmitt MP, Holmes RK. Iron-dependent regulation of diphtheria toxin and siderophore expression by the cloned *Corynebacterium diphtheriae* repressor gene *dtxR* in *C. diphtheriae* C7 strains. Infect Immun. 1991;59(6):1899-904.

7. Oram DM, Jacobson AD, Holmes RK. Transcription of the contiguous *sigB*, *dtxR*, and *galE* genes in *Corynebacterium diphtheriae*: evidence for multiple transcripts and regulation by environmental factors. J Bacteriol. 2006;188(8):2959-73.

8. Schafer A, Tauch A, Jager W, Kalinowski J, Thierbach G, Puhler A. Small mobilizable multi-purpose cloning vectors derived from the *Escherichia coli* plasmids pK18 and pK19: selection of defined deletions in the chromosome of C*orynebacterium glutamicum*. Gene. 1994;145(1):69-73.

9. Drazek ES, Hammack CA, Schmitt MP. *Corynebacterium diphtheriae* genes required for acquisition of iron from haemin and haemoglobin are homologous to ABC haemin transporters. Mol Microbiol. 2000;36(1):68-84.

10. Bibb LA, Schmitt MP. The ABC transporter HrtAB confers resistance to hemin toxicity and is regulated in a hemin-dependent manner by the ChrAS two-component system in *Corynebacterium diphtheriae*. Journal of bacteriology. 2010;192(18):4606-17.
